# Alumi Association of Albany Medical College

**Published:** 1874-01

**Authors:** 


ALUMNI ASSOCIATION OF ALBANY MEDICAL COLLEGE.—At the commencement exercises of the Albany Medical College, January 20th, an Alumni Association was organized. About eighty names were registered, and the following officers we elected.

*President*—Dr. H. D. Didama, Syracuse, ('46).

*For Vice-President*—Dr. T. B. Reynolds, Saratoga Springs, ('42); Dr. J. M. Schemerhorn, Stockport, ('45); Dr. John Swinburne, Albany, ('46); Dr. A. Van Woert, Visscher's Ferry, ('46); Dr. H. Lenardson, Charleston Four Corners, (47).

*For Secretary*—Dr. W. G. Tucker, Albany, ('70).

*For Treasurer.*—Dr. James S. Bailey, Albany, ('53).

*For Members of Executive Committee*—Dr. Levi Moore, Albany ('51); Dr. H. March, Albany, ('53); Dr. H. B. Whiton Troy, ('54); Dr. R. Loughran, Kingston, ('57); Dr. H. B. Mahen, Ilion, ('57); Dr. C. A. Winship, Eagle Mills, (58); Dr. N. M. Carter, Poughkeepsie, ('59).

We hope that all graduates of the Albany Medical College will give the Association their hearty support.

